# Ensemble of Multiple Classifiers for Multilabel Classification of Plant Protein Subcellular Localization

**DOI:** 10.3390/life11040293

**Published:** 2021-03-30

**Authors:** Warin Wattanapornprom, Chinae Thammarongtham, Apiradee Hongsthong, Supatcha Lertampaiporn

**Affiliations:** 1 Applied Computer Science Program, Department of Mathematics, Faculty of Science, King Mongkut’s University of Technology Thonburi, Bangkok 10140, Thailand; warin.wat@mail.kmutt.ac.th; 2 Biochemical Engineering and Systems Biology Research Group, National Center for Genetic Engineering and Biotechnology, National Science and Technology Development Agency at King Mongkut’s University of Technology Thonburi, Tha Kham, Bang Khun Thian, Bangkok 10150, Thailand; chinae@biotec.or.th (C.T.); apiradee@biotec.or.th (A.H.)

**Keywords:** ensemble machine learning, plant protein, feature extraction, feature selection, go term, consensus voting, average voting, subcellular localization prediction

## Abstract

The accurate prediction of protein localization is a critical step in any functional genome annotation process. This paper proposes an improved strategy for protein subcellular localization prediction in plants based on multiple classifiers, to improve prediction results in terms of both accuracy and reliability. The prediction of plant protein subcellular localization is challenging because the underlying problem is not only a multiclass, but also a multilabel problem. Generally, plant proteins can be found in 10–14 locations/compartments. The number of proteins in some compartments (nucleus, cytoplasm, and mitochondria) is generally much greater than that in other compartments (vacuole, peroxisome, Golgi, and cell wall). Therefore, the problem of imbalanced data usually arises. Therefore, we propose an ensemble machine learning method based on average voting among heterogeneous classifiers. We first extracted various types of features suitable for each type of protein localization to form a total of 479 feature spaces. Then, feature selection methods were used to reduce the dimensions of the features into smaller informative feature subsets. This reduced feature subset was then used to train/build three different individual models. In the process of combining the three distinct classifier models, we used an average voting approach to combine the results of these three different classifiers that we constructed to return the final probability prediction. The method could predict subcellular localizations in both single- and multilabel locations, based on the voting probability. Experimental results indicated that the proposed ensemble method could achieve correct classification with an overall accuracy of 84.58% for 11 compartments, on the basis of the testing dataset.

## 1. Introduction

Subcellular localization is one of the key properties considered in the functional annotation of proteins [1,2,3]. Identifying the subcellular locations of proteins is immensely helpful for understanding their function and designing or identifying drug targets. Predicting the subcellular locations of proteins in cells is an important step for providing useful insights into protein functions and the mechanisms underlying various biological processes. Knowledge of protein localization might also provide valuable information for target identification for drug discovery [4,5]. With the advent of high-throughput sequencing technology, innumerable protein sequences are now being progressively identified and submitted to public sequence databases [3]. According to the statistical release for 2020 (Release: 2020_06 of 2 December 2020), UniProtKB contains 59,932,518 sequence entries, but only 350,510 of the proteins have a reviewed subcellular localization status (manually annotated) [6]. Due to the huge amount of generated data, biochemical wet-lab experiments for locating proteins are particularly laborious and costly, involving time-consuming, labor-intensive tools that require a large number of skilled researchers [7,8]. Hence, there is a need for an accurate alternative computational method that utilizes the capabilities of artificial intelligence and machine learning, to provide fast and accurate results for identifying new proteins [9]. 

Diverse subcellular localization computational prediction tools were proposed using different training data procedures, data features, and machine learning algorithms [10,11,12,13,14,15,16,17,18,19,20,21,22,23,24,25,26,27,28,29,30,31,32]. Some tools used the support vector machine (SVM) algorithm such as MultiLoc2 [10], Plant-mSubP [11], mGOASVM [12], WegoLoc [13], and LocTree [14]. Some tools used the K-nearest neighbors (KNN) algorithm, such as Plant_mPLoc [15] and iLoc-Plant [16]. Some tools used naïve Bayes such as Yloc [17] and ngLOC [18]. Some tools applied neural network algorithms such as SCLpredT [19], and some applied deep learning, such as DeepPSL [20]. In addition to differences in the algorithm architecture, these tools also differ in the features used to train the model. 

The existing methods can be roughly classified into three main feature groups—the first type of feature is based on a comparative or homology approach utilizing the advantages of the gene ontology (GO) term vocabulary approach. iLoc-Plant [16], Plant_mPLoc [15], pLoc-mEuk [21], mGOASVM [12], WegoLoc [13], LocTree [14], YLoc [17], and HybridGO-Loc [22] use the GO term as the main feature. However, using solely this type of feature might not be suitable for newly discovered sequences with no known homologous sequences in the database. The second type of feature involves searching for the presence of motifs associated with a known biochemical function. For example, BUSCA [23] and localizer [24] use sorting signal peptide-based features as the main features. 

The third type of feature is the functions or characteristics of the protein sequences using their physicochemical and amino acid composition-related characteristics. Examples of tools that used this type of feature are ngLOC [18], which is solely based on the sequence-based feature and those in [25,26,27]. In conclusion, these tools differ in three critical aspects—trained model, trained features, and number of coverage locations. 

For the subcellular location problem, there are many challenges. (1) The type of organism has different numbers of locations. (2) There is a limited of number of trained data in some locations. (3) The subcellular localization problem is a multi-classification problem in which some localizations can be predicted with high accuracy because there are more sample data and explicit features to describe them, whereas other locations are limited in the known number of sequences and characteristics, resulting in variations in accuracy performance between localizations. (4) There is a multi-label problem in which many proteins are located in multiple locations. There is evidence indicating that proteins with multiple locations might present specific biological functions that are intriguing to investigators [31]. Therefore, it is necessary to explore features and appropriate classification methods for improving prediction performance [32].

For the plant subcellular localization classification problem, only a few methods were designed and developed, especially for predicting the multi-label subcellular localization of plant proteins [7,11]. The number of subcellular locations in plants is large, and the number of proteins in each location is different or imbalanced. Most methods for plant are SVM or KNN.

In this research, we contribute to (1) building an ensemble machine learning classifier that aggregates various diverse individual classifiers, specifically for addressing the problem of subcellular localization in plants, which is considered a multiclass, multilabel classification problem. Ensembles can be more generalized [33], can help reduce the risk of overfitting [34] and can ultimately provide stable, accurate prediction results, by combining the strengths and reducing the weakness of diverse individual classifiers. Moreover, an ensemble of different classifiers with appropriate combinations has the benefit of being able to learn nonlinear or complex boundary problems [35].

We also (2) explore and extract as many features involved in plant localization as possible, to characterize the sequences of plant proteins. There are weaknesses and strengths of different features. Therefore, we use different feature selection methods to find the effective set of discriminative and nonredundant features that plays a critical role in predicting the subcellular location of plant proteins. (3) An increasing number of multiple-localization proteins have a significant function in the cell. The proposed method also provides a distribution of prediction score that indicates the probability that a protein might reside in multiple compartments and provides additional information about the GO term involved.

## 2. Materials and Methods

**Figure 1:**
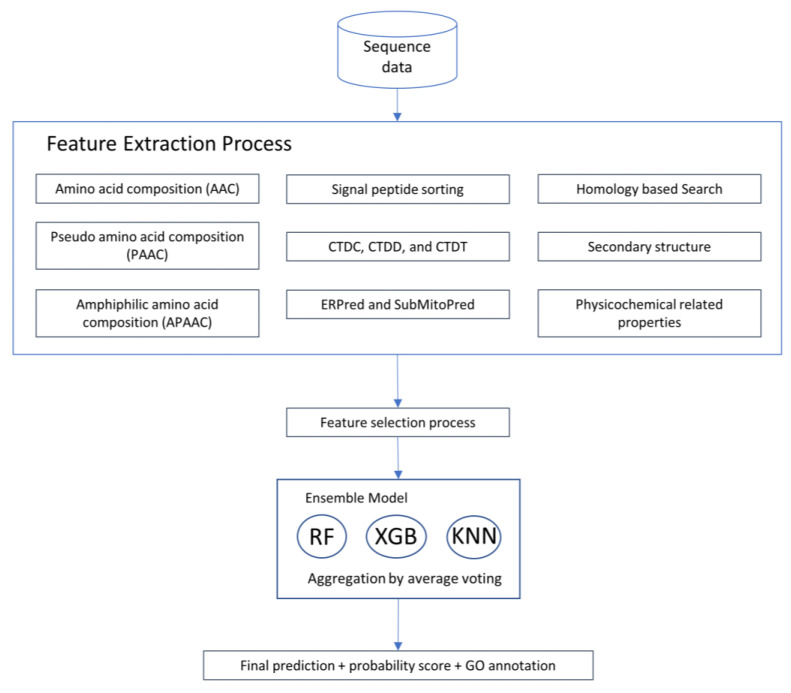
Workflow of the program.

There are key steps in the workflow, as shown in Figure 1. (1) Collect data; (2) extract various features, including sequence feature-based, homology-based, signal peptide- based, and physicochemical property-related features; (3) select a subset of features with effective discrimination power; and (4) develop and test the prediction performance of the ensemble classifier.

### 2.1. Dataset

A training and testing dataset obtained from Plant-mSubP [11] was used to train and evaluate the performance of the program for 11 protein locations. These data were already filtered according to the criterion of <30% similarity, using the BLASTclust, as described in [11]. In this work, to reduce the high imbalance observed for plastids and cell membranes versus the remaining locations, we applied CD-HIT [36], with a similarity cutoff of <25%, followed by random subsampling for the proteins in the plastid and cell membrane locations. A summary of the number of proteins in the training and testing datasets is shown in Table 1. The training and independent testing sequence dataset is also provided in Supplementary Dataset S1.

### 2.2. Feature Extraction

In this work, we extracted various types of features to represent a protein in a vector of 479 features. Table 2 summarizes the features and abbreviations used in this work. The features could be grouped into 7 main types as follows:

(i) Sequence based features: The amino acid composition of the entire amino acid sequence and Chou’s pseudo amino acid composition (PseAAC) in various modes were generated—pseudo amino acid composition (PseAAC) in parallel and in series correlations. Chou’s PseAAC [37] is widely used to convert complicated protein sequences with various lengths to fixed-length numerical feature vectors that incorporate sequence-order information. Compared to AAC, PseAAC is more informative, and can represent a protein sequence and incorporate its sequence-order information. Hence, it is widely applied for prediction in various amino acid sequence-based prediction problems [38]. PseACC was calculated using the Pse-in-one program [39] with parameter lambda = 2, 10 and weight = 0.05, 0.1.
(ii) Composition–transition–distribution (CTD): Three types of descriptors based on the grouped amino acid composition [40,41] (composition (CTDC), transition (CTDT) and distribution (CTDD) descriptors) were calculated. CTD was calculated using the protr R package [42,43]. All amino acid residues were divided into 3 groups—neutral, hydrophobic, and polar—according to 7 types of physicochemical properties, as defined in [41]. The 7 physicochemical properties used for calculating these features were hydrophobicity, normalized van der Waals volume, polarity, polarizability, charge, secondary structures, and solvent accessibility.
(iii) Various physicochemical property-based features: Quasi-sequence-order descriptors (QSO) [44], crucian properties [45], zScales [46], FASGAI vectors (factor analysis scales of generalized amino acid information) [47], tScales [48], VHSE-scales (principal components score vectors of hydrophobic, steric, and electronic properties) [49], protFP [50], stScale [51], MS-WHIM score [52], the aliphatic index [53], the autocovariance index [53], the Boman (potential protein interaction) index [54], the net charge, cross-covariance index [45], instability index [55], the hydrophobic moment, and the isoelectic point (pI) were calculated using the peptide R package [56] with parameter nlag = 10 and weight = 0.1.
(iv) Signal peptide-based features: In addition to the sequence features mentioned above, functional or signal peptide regions were used in this prediction. The signal peptide was associated with the transfer to or function of a protein in its localization site [57]. Nuclear localization signals (NLSs) were used as important features for detecting nuclear proteins. For example, a protein containing a signal peptide is likely to be transferred to the secretory pathway, while a protein containing an NLS is likely to be localized in the nucleus. In this work, to identify the signal sequences for the secretory pathway (signal peptides) and predict the positions of the signal peptide cleavage sites and transmembrane, the prediction scores obtained from well-known prediction programs, such as TargetP [58], SignalP [59], Phobius [60], and TMHMM [61], were used as feature scores (features: SP, cTP, mTP, other, and TM). The NLS was predicted using the Hidden Markov Models (HMMs) of NLStradamus [62] to predict the NLSs of the sequences (feature: NLS). However, there are some limitations of this type of feature; i.e., the signal peptide is not yet completely understood, and the set of currently known signals might be incomplete.
(v) Integration of other methods: We used the ERPred [63] Score and SubMito [64] SVM scores as features for discriminating ER and mitochondrial proteins, respectively. These programs were not used directly to predict locations. However, they were used to generate the numerical feature to complement each other as parts of the model to learn in making decisions.
(vi) Secondary structure conformation features: The aggregation, amyloid, turn, alpha-helix, helical aggregation, and beta-strand conformation secondary structures were calculated using the Tango program [65].
(vii) Homology and Gene Ontology (GO) annotation-based features: BLAST [66] was used to search for homologous sequences. This feature is highly effective when a homologous protein with a localization annotation is available. Evolutionarily, closely related proteins present a high probability of showing similar subcellular localizations. Therefore, this type of feature can outperform other features when a homologous protein with a localization annotation is available [67]. However, there is also a limitation of this type of feature, where no homology is found between the query and target sequence. The performance of sequence homology-based methods might be significantly reduced when homologous sequences are not detected [68]. However, using the GO feature can result in a noisy and confound prediction [69,70] in the case when a protein could have multiple GO terms that map to different subcellular localizations, resulting in inconsistency with the true subcellular locations of proteins [12]. The GO database used in this work is a compact database that was filtered to remove redundant information (<25% sequence similarity threshold) and contained only representative sequences that did not overlap in the training and testing data. A set of GO terms in the “cellular component” category was retrieved by searching against the Gene Ontology Annotation database [71] and the UniProtKB/Swiss-Prot database [72]. The GO terms used in this work are summarized in Table 3.

### 2.3. Feature Selection

We reduced the number of features and identified optimal feature subsets using various types of feature selection methods, such as ReliefF [73], OneR [74], and correlation-based feature selection (CFS) [75] with a genetics search.

### 2.4. Model Selection

The application of the K-nearest neighbor (KNN) is a well-known nonparametric technique used in statistical pattern classification, owing to its simplicity, intuitiveness, and effectiveness [76]. The basic idea of this rule is that an unclassified object is assigned to the class represented by a majority of its k nearest neighbors in the training set. When different values of k were compared, we found that the optimal value of k = 12 with inverse weighting, yielded the optimal classification performance for this problem. Thus, the KNN with K = 12 and inverse weighting was used thereafter. Note that KNN is robust to datasets with imbalanced classes and multimodal distributions. 

The random forest (RF) algorithm is one of the most commonly used bagging ensemble algorithms because of its flexibility and ease of use. This algorithm can produce good results without hyperparameter tuning. The RF approach is an ensemble technique with the ability to achieve high accuracy and prevent overfitting, by making use of voting in multiple decision trees. (RF parameter: no. estimators = 100).

The extreme gradient boosting (XGB) algorithm is a gradient boosting ensemble algorithm. The boosting algorithm adjusts the weights according to a differential loss function and then uses the adjusted weights in the next training iteration. (params: no. estimators (nrounds) = 50, max_depth = 5, eta = 0.1, eval_metric = “mlogloss”, num_class = 11). 

A heterogeneous ensemble classification model combining the 3 different classifier algorithms is obtained through aggregation to increase the performance of the model. In this work, we used the average voting result among individual classification models as a consensus score of the heterogeneous ensemble model.

We adopted the 10-fold cross-validation method to investigate the classification performance based on training data. 

Based on the 10-fold cross-validation, the feature selection and model selection processes were performed using only the training dataset. Then, the best-performing model was selected and later used as the prediction model of the program. The test dataset was used independently to evaluate the selected models and benchmarked against the competitive programs. The ensemble-PlantSCL standalone program and classification model is available to download at http://www.ncrna-pred.com/ensemblePSCL.htm (accessed on 30 March 2021).

### 2.5. Evaluation Measurement

To evaluate the classification performance of the model, the following metrics were used:

(1) $$\mathrm{ACC}=\frac{\mathrm{TP}+\mathrm{TN}}{\left(\mathrm{TP}+\mathrm{TN}+\mathrm{FP}+\mathrm{FN}\right)}$$

(2) $$\mathrm{Sn}=\frac{\mathrm{TP}}{\left(\mathrm{TP}+\mathrm{FN}\right)}$$

(3) $$\mathrm{Sp}=\frac{\mathrm{TN}}{\left(\mathrm{TN}+\mathrm{FP}\right)}$$

(4) $$\mathrm{MCC}=\frac{\;\mathrm{TP}\times \mathrm{TN}-\mathrm{FP}\times \mathrm{FN}}{\sqrt{\left(\mathrm{TP}+\mathrm{FP}\right)\left(\mathrm{TP}+\mathrm{FN}\right)\left(\mathrm{TN}+\mathrm{FP}\right)\left(\mathrm{TN}+\mathrm{FN}\right)}\;}$$

where ACC, Sn, Sp, and MCC are the accuracy, sensitivity, specificity, and Matthews coefficient correlation, respectively. These measurements were calculated based on the numbers of true positives (TPs), true negatives (TNs), false positives (FPs), and false negatives (FNs). The area under the receiver operating characteristic (ROC) curve (AUC) was calculated to assess the tradeoff between the sensitivity and specificity performance of the different methods. The ROC curve is a plot of the TP vs. FP rates at different thresholds. For a perfect predictor, the AUC is equal to 1.

## 3. Results and Discussion

### 3.1. Comparison of Different Features/Feature Analysis

**Figure 2:**
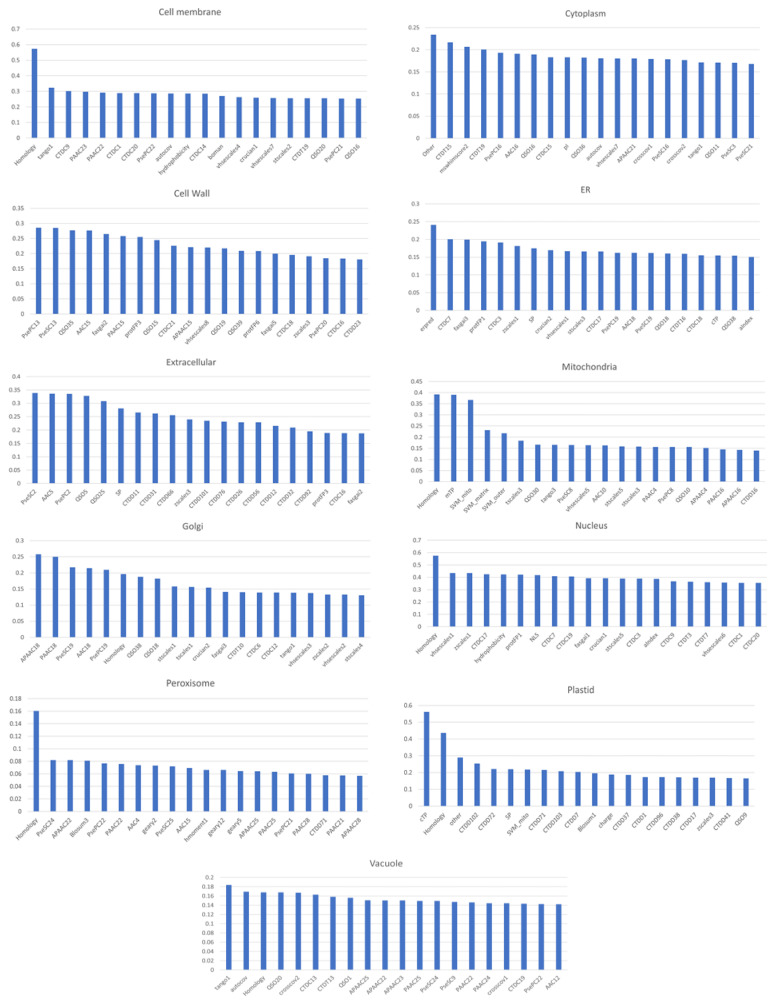
Top 20 features that are highly correlated with each localization target.

To detect hidden patterns, in this work, we utilized various types of features, such as homology-based, sequence-based, signal-based, and physicochemical property-related features, to represent peptides with a vector of 479 total features. We tried to collect and extract as many known subcellular localization-related features as possible, so that the number of features was sufficient to explain the characteristics of various subcellular localizations. The evaluation of subcellular localization is a difficult problem due to its multilabel and multiclass nature. We are, therefore, interested in exploring which features show a high correlation with particular localizations. We generated 11 datasets of one localization-vs-other localizations (1-vs-All-1 locations) from the training dataset and then calculated the Pearson correlation coefficient (PCC) analysis. The top 20 features showing the highest correlation with each location were plotted, as shown in Figure 2, to display the features that contributed the most to each location. We found a moderate correlation (PCC of approximately 0.5) between features and target locations, such as plastids and nuclei. The proteins at these locations exhibit specific informative or signal features that allow them to be discriminated from others. However, in many locations, such as peroxisomes and vacuoles, there is quite a low correlation (PCC < 0.2) between the top features and the target location.

### 3.2. Discriminative and Informative Reduced Feature Subset

As shown in Figure 2, no single feature was found among the top features in all 11 localizations. The efficient feature representation of a protein sequence is a very important aspect of subcellular localization. However, a group of multiple features might include irrelevant and redundant features and can therefore cause high dimensionality. High-dimensional features that include much redundant information might harm and negatively influence the performance of the classifier. Dimensionality reduction algorithms can help to eliminate redundant data from the original feature space and are widely used in machine learning [32]. Therefore, a feature-selection step is needed to identify discriminative and nonredundant feature subsets that can discriminate all locations. To select the discriminative feature subset, a comparison of the empirical performance of individual predictive models using different feature sets from various feature-selection methods, such as ReliefF, OneR, and CFS, was performed, as shown in Table 4. The OneR, ReliefF, and CFS feature sets contained 87, 95, and 109 features, respectively (listed in Supplementary Tables S1–S3).

Model performance decreased slightly for the model incorporating all features, while model performance increased significantly for the model with feature selection subsets. Divergence in the accuracy of individual models (RF, KNN, XGB) was observed when using different feature-selection approaches. Moreover, there were improvements in the accuracy of individual models when feature selection was used. Notably, the accuracy of the heterogeneous ensemble model using feature subsets (HeteroEnsembles 1, 2, and 3) increased by approximately 2.76–3.68%, as compared to the ensemble model that used all features.

### 3.3. A 10-Fold Cross-Validation of Predictive Performance with the Training Dataset

The experiments were conducted via 10-fold cross-validation of the training process and were validated by independent testing datasets. To investigate the classification performance of different models and different feature subsets, the feature subset was applied as input vectors for the classifier, KNN, RF, XGB, and ensemble models, followed by evaluation and comparison in three different optimal sets of feature subsets. Thereafter, the individual classifiers (KNN, RF, and XGB) were aggregated through average voting to obtain the final prediction of a heterogeneous ensemble model. According to Table 4, based on the performance obtained with the training data, the HeteroEnsemble3 model, which is an ensemble of the RF, KNN, and XGB models obtained using the CFS feature subset, showed the highest ACC, MCC, and AUC values of 94.68%, 0.94, and 0.997, respectively, among the three ensemble models. The ensemble model significantly improved classification performance over that was obtained with the individual KNN, RF, and XGB classifiers, in terms of the classification accuracy and AUC. The high AUC value (0.997) implies that the ensemble method achieves better predictive balance among the 11 subcellular locations. Therefore, the HeteroEnsemble3 model was selected for further use.

### 3.4. Classification Performance for the Independent Testing Dataset

To assess the performance of the method, evaluation had to be carried out with an independent testing dataset that was not used during the training step. When tested with the independent data, accuracies of 85.47%, 84.58%, and 70.27% were achieved for single-label, single-, and dual-label, and dual-label proteins, respectively, as shown in Table 5. Compared to the performance of Plant_msubP (64.36%, 64.84%, and 81.08%) reported in [11], our model showed improvements in accuracy for the single-label, single-, and dual-label datasets. In addition to the percent of correctly predicted results in each location, which reflects only the true positive, we calculated the MCC values by using all four values from the confusion matrix (true positive, true negative, false positive, and false negative). MCC is a more reliable statistical measurement [77]. A high value close to 1 means that the prediction is good in all four confusion matrix categories, proportional to both the size of positive samples and the size of negative samples in the dataset. The detailed classification result with prediction probability is provided in Supplementary Table S4.

As shown in Table 5, we also found that the model had difficulty distinguishing some locations, such that the performance of our method for vacuole and peroxisome proteins was not good. There were many misclassifications in some important subcellular compartments, such as the Golgi and peroxisome. Many Golgi apparatus proteins were misclassified as being located in the endoplasmic reticulum (ER). Three misclassified peroxisome proteins were classified as being located in the cytoplasm, nucleus, and cell membrane. Low accuracy was observed for certain compartments, such as the vacuoles and peroxisomes, which was consistent with the results of our feature analysis, shown in Figure 2, indicating quite low correlations (PCC < 0.2) between the top features and the locations of peroxisomes and vacuoles. However, the method was still effective in its predictions for the 11 subcellular compartments overall, as shown in Table 5.

### 3.5. Comparison with Other Existing Tools 

Using the independent testing dataset, we could compare our method with the available prediction tools that also support multilocation prediction, based on the results reported in [11]. As shown in Table 6, our method achieved 84.58% accuracy for single- and multi-localization data together, and 70.27% accuracy for multi-localization testing data alone, thus significantly outperforming the other methods in both cases.

In summary, our ensemble predictor achieved good prediction results for most of the 11 localization datasets, which sufficiently demonstrated that the ensemble prediction method incorporated with the selected feature subset proposed in this paper was accurate, consistent, and robust/stable.

## 4. Conclusions

In this work, efforts were made to collect various informative features and to develop heterogeneous ensemble methods for both single- and multiple-location proteins, to address the problem of subcellular localization. Several features were included in this approach to represent proteins, such as the amino acid composition, pseudo amino acid composition, annotation-based methods (GO-based features), and sorting signals (signal and transit peptides, transmembrane domains). To include more divergence in the model to increase the stability of our heterogeneous ensemble model, we built the heterogeneous ensemble model, based on aggregation by average voting, based on KNN, RF, and XGB. Through a proper combination of the diverse predictors via an appropriate fusion method, the ensemble predictor could proficiently exploit the strength and reduce the weakness of any component predictor. The proposed ensemble predictor provided an efficient final decision based on average voting to make final predictions for 11 plant-localization datasets. In addition, the method improved protein subcellular localization classification by integrating various informative features so that the various protein properties could be considered from multiple points of view, to obtain a more accurate and robust/stable prediction. The method achieved 84.58% accuracy in its predictions for 11 localizations in plants, with the ability to classify multiplex proteins/multi-localization proteins, and provide GO term annotations.

## Figures and Tables

**Table 1:** Number of proteins from each location in the training and testing datasets.

Type	Subcellular Location	Training Data (Original)	Training Data (25% CD-HIT)	Testing Data
Single location	Plastid	2468	533	248
	Cytoplasm	351	351	40
	Extracellular	140	140	14
	Nucleus	568	568	63
	Mitochondrion	447	447	52
	Cell membrane	829	438	92
	Golgi Apparatus	204	204	23
	Endoplasmic reticulum	280	280	29
	Vacuole	176	176	20
	Peroxisome	57	57	6
	Cell wall	37	37	5
Multilocation	Mito-Plastid	118	118	13
	Cyto-Nucleus	170	170	20
	Cyto-Golgi	34	34	4
**Total**		**5879**	**3553**	**629**

**Table 2:** Summary of features or descriptors that were used in this research.

Features (Total = 479 Features)	Abbreviation
Amino acid Composition	AAC1-AAC20
Amphiphilic PseAAC	APAAC1-APAAC30
BLOSUM matrix-derived	Blosum1-Blosum8
Composition descriptor of the CTD	CTDC1-CTDC21
Distribution descriptor of the CTD	CTDD1-CTDD105
Transition descriptor of the CTD	CTDT1-CTDT21
Geary autocorrelation	Geary1-Geary40
Pseudo amino acid composition	PAAC1-PAAC30
Parallel pseudo amino acid composition	PsePC1-PsePC22
Serial pseudo amino acid composition	PseSC1-PseSC26
Net charge	Charge
Potential protein interaction index	Boman
Aliphatic index of protein	aIndex
Autocovariance index	autocov
Crosscovariance1	Crosscov1
Crosscovariance2	Crosscov2
Cruciani covariance index	Crucian1-Crucian3
Factor analysis scales of generalized amino acid information	fasgai1-fasgai6
Hmoment alpha helix	Hmomonet1
Hmoment beta sheet	Hmoment2
Hydrophobicity index	hydrophobicity
Instability index	Instaindex
MS-WHIM scores derived from 36 electrostatic potential properties	mswhimscore1-mswhimscore 3
Isoelectric point (pI)	pI
Average of protFP	protFP1-protFP8
ST-scale based on physicochemical properties	stscales1-stscales8
T-scale based on physicochemical properties	tscales1-tscales5
VHSE-scale based on physicochemical properties (vhsescales1	vhsescales1-vhsescales8
Z-scale based on physicochemical properties	stscales1-stscales5
Quasi-sequence-order descriptor	QSO1-QSO60
Sequence-order-coupling numbers	SOCN1-SOCN20
Chloroplast transit peptide	cTP
Mitochondrial transit peptide	mTP
Signal peptide cleavage site score	SP
Number of predicted transmembrane segments	TM
Other location score from targetP	other
Nuclear localization signal	NLS
SVM score from Erpred	erpred
SubmitoPred (SVM_score_mito)	SVM_mito
SubmitoPred (SVM_inner_mem)	SVM_mem
SubmitoPred (SVM_inter_mem)	SVM_inter
SubmitoPred (SVM_score_matrix)	SVM_matrix
SubmitoPred (SVM_score_outer_mem)	SVM_outer
Aggregation (tango1)	Tango1
Amyloid (tango2)	Tango2
Turn-turns (tango3)	Tango3
Alpha-helices (tango4)	Tango4
Helical aggregation (tango5)	Tango5
Beta-strands (tango6)	Tango6
Homology based feature (GO term)	Homology

**Table 3:** Summary of cellular component GO terms used in this work.

Go term; ‘Cellular component’
GO:0005737; cytoplasm
GO:0005783; endoplasmic reticulum
GO:0005788; endoplasmic reticulum lumen
GO:0005789; endoplasmic reticulum membrane
GO:0005793; endoplasmic reticulum-Golgi intermediate compartment
GO:0005615; extracellular space
GO:0005794; Golgi apparatus
GO:0005796; Golgi lumen
GO:0000139; Golgi membrane
GO:0005739; mitochondrion
GO:0005740; mitochondrial envelope
GO:0005743; mitochondrial inner membrane
GO:0005758; mitochondrial intermembrane space
GO:0005759; mitochondrial matrix
GO:0031966; mitochondrial membrane
GO:0005741; mitochondrial outer membrane
GO:0005886; plasma membrane
GO:0005618; cell wall
GO:0005634; nucleus
GO:0009536; plastid
GO:0009528; plastid inner membrane
GO:0005777; peroxisome
GO:0005778; peroxisomal membrane
GO:0005773; vacuole
GO:0005774; vacuolar membrane
GO:0016020; membrane
GO:0009507; chloroplast

**Table 4:** Classification training performances for different feature subsets.

**All Features (479)**	**RF**	**KNN**	**XGB**	**HeteroEnsemble**
ACC	82.72%	82.62%	85.97%	91.00%
MCC	0.795	0.798	0.845	0.896
AUC	0.977	0.897	0.975	0.993
**OneR (87)**	**RF**	**KNN**	**XGB**	**HeteroEnsemble1**
ACC	92.02%	91.51%	93.87%	93.76%
MCC	0.907	0.902	0.932	0.929
AUC	0.995	0.991	0.993	0.996
**ReliefF (95)**	**RF**	**KNN**	**XGB**	**HeteroEnsemble2**
ACC	94.27%	89.57%	93.46%	93.97%
MCC	0.935	0.879	0.928	0.932
AUC	0.996	0.991	0.992	0.996
**CFS + Genetics (109)**	**RF**	**KNN**	**XGB**	**HeteroEnsemble3**
ACC	94.48%	93.05%	95.30%	94.68%
MCC	0.938	0.921	0.948	0.94
AUC	0.996	0.994	0.996	0.997

**Table 5:** Classification performance of the heterogeneous ensemble for the independent testing dataset.

Type	Subcellular Location	Testing Data	Correctly Predicted	Percent	MCC
Single location	Plastid	248	238	95.97%	0.756
	Cytoplasm	40	34	85%	0.829
	Extracellular	14	9	64.28%	0.756
	Nucleus	63	61	96.82%	0.854
	Mitochondrion	52	31	59.61%	0.708
	Cell membrane	92	81	88.04%	0.792
	Golgi Apparatus	23	14	60.86%	0.747
	Endoplasmic reticulum	29	25	86.21%	0.710
	Vacuole	20	5	25%	0.359
	Peroxisome	6	3	50%	0.705
	Cell wall	5	5	100%	1
	**Total (Single location)**	**592**	**506**	**85.47%**	**0.747**
Multilocation	Mito-Plastid	13	8	61.54%	0.607
	Cyto-Nucleus	20	18	90%	0.897
	Cyto-Golgi	4	0	0%	0
	**Total (multilocation)**	**37**	**26**	**70.27%**	**0.501**
**Total All**		**629**	**532**	**84.58%**	**0.694**

**Table 6:** Comparison of prediction accuracy for an independent dataset with the accuracy of existing tools that support multiple-labels localizations. The actual accuracy is calculated as a percentage of the ratio of the number of correctly predicted sequences divided by the total number of sequences in the independent dataset.

Method	Machine Learning Technique	Accuracy (Single + Dual Label Data)	Accuracy (Dual Label Data)
YLoc [17]	Naïve Bayes	34.35	35.89
Euk-mPloc 2.0 [28]	OET-KNN ^1^	53.5	44.86
iLoc-Plant [16]	ML-KNN ^2^	37.42	34.42
Plant-mSubP [11]	SVM ^3^	64.84	81.08
Our model	Ensemble	84.58	70.27

^1^ OET-KNN = Optimized Evidence-Theoretic K-Nearest Neighbor; ^2^ SVM = Support Vector Machine; ^3^ ML-KNN = Multi-labeled K-Nearest Neighbor.

## Data Availability

The data presented in this study are available in the supplementary files.

## Supplementary Materials

The following are available online at https://www.mdpi.com/article/10.3390/life11040293/s1. Table S1: Feature Subset 1 Selected by OneR, Table S2: Feature Subset 2 Selected by ReliefF with number of neighbors = 10, Table S3: Selected by CFS with genetics search (number of generations = 1000, number of populations = 200, mutation rate = 0.013, crossover = 0.6). Selected features: 109 features, Table S4: The detailed classification result with prediction probability.
