# Supplementary material for: Ensemble of Multiple Classifiers for Multilabel Classification of Plant Protein Subcellular Localization
Source: Life (Basel). 2021 Mar 30;11(4):293. doi: 10.3390/life11040293 (PMC8066735; doi:10.3390/life11040293)
Supplement: Supplementary file 1 [file life-11-00293-s001.zip › life-1110858-supplementary.docx]

Supplementary of Ensemble of Multiple Classifiers for Multilabel Classification of Plant Protein Subcellular Localization

**Table S1.** Selected by OneR. Selected features: 87 features

| **Homology** | **CTDC14** | SVM_inter | tscales4 | **PAAC22** |
| --- | --- | --- | --- | --- |
| **NLS** | **crucian1** | QSO31 | QSO29 | **AAC4** |
| **hydrophobicity** | **CTDT3** | instaindex | CTDT9 | **PseSC25** |
| **vhsescales1** | vhsescales4 | APAAC5 | QSO14 | QSO6 |
| **SVM_mito** | **QSO35** | **PseSC8** | APAAC1_2 | **CTDT15** |
| **zscales1** | **mTP** | **QSO25** | PsePC4 | **stscales2** |
| **boman** | **CTDC1** | stscales7 | QSO27 | **vhsescales5** |
| **cTP** | **CTDC20** | **APAAC18** | **AAC10** |  |
| **CTDC19** | **CTDT7** | APAAC7 | **zscales2** |  |
| **protFP1** | QSO34 | **mswhimscore2** | **CTDC15** |  |
| **SP** | **CTDC9** | **QSO10** | **CTDT16** |  |
| fasgai1 | **SVM_matrix** | APAAC9 | **PsePC8** |  |
| **tango1** | **CTDC13** | **SVM_outer** | **CTDD72** |  |
| **CTDC3** | **Erpred** | **QSO36** | **vhsescales7** |  |
| **other** | QSO24 | **CTDD71** | **CTDT13** |  |
| **stscales5** | CTDT1 | **QSO39** | QSO37 |  |
| QSO26 | **vhsescales6** | QSO40 | **QSO38** |  |
| CTDC17 | APAAC1_6 | fasgai4 | **vhsescales8** |  |
| **aIndex** | **QSO11** | tango5 | **Blosum3** |  |
| **CTDC7** | CTDT21 | **CTDC18** | **PseSC24** |  |

**Table S2.** Selected by ReliefF with number of neighbors = 10. Selected features: 95 features

| **Homology** | **CTDC9** | **PseSC3** | PsePC3 | PAAC20 |
| --- | --- | --- | --- | --- |
| TM | **AAC18** | PseSC4 | **CTDD72** | APAAC9 |
| **other** | **PsePC19** | **APAAC22** | **PseSC24** | CTDT1 |
| **cTP** | **CTDC1** | **CTDC13** | fasgai4 | AAC3 |
| **SP** | **CTDC20** | **stscales3** | **mswhimscore2** | PsePC12 |
| **mTP** | **fasgai1** | **CTDC19** | **fasgai3** | CTDT9 |
| **NLS** | **QSO18** | **crucian2** | Pse_PC4 | AAC14 |
| **SVM_matrix** | **CTDC15** | **vhsescales6** | **CTDC18** | crucian3 |
| **Erpred** | **Blosum3** | **vhsescales5** | **PsePC8** | PAAC9 |
| **SVM_mito** | **crucian1** | **AAC4** | AAC11 | **stscales1** |
| **tango1** | **CTDT15** | **AAC10** | tscales4 | **tscales3** |
| **protFP1** | **boman** | **CTDT19** | **QSO30** | PseSC12 |
| **vhsescales1** | **QSO38** | CTDT13 | **crosscov1** | CTDT8 |
| **SVM_outer** | **PAAC18** | CTDT2 | **zscales2** | **CTDD102** |
| **stscales5** | **CTDT3** | mswhimscore1 | **autocov** | **tscales1** |
| **hydrophobicity** | **CTDT7** | **PseSC8** | **Blosum1** |  |
| **CTDC7** | **CTDC3** | PseSC10 | **APAAC21** |  |
| **PseSC19** | **APAAC18** | AAC6 | **PseSC25** |  |
| **zscales1** | **CTDC14** | SVM_inter | mswhimscore3 |  |
| **CTDC17** | **aIndex** | **geary2** | APAAC20 |  |

**Table S3.** Selected by CFS with genetics search (number of generations = 1000, number of populations = 200, mutation rate = 0.013, crossover = 0.6).Selected features: 109 features

| AAC2 | CTDD18 | PAAC10 | **fasgai1** | **PsePC8** | **mTP** |
| --- | --- | --- | --- | --- | --- |
| AAC13 | CTDD22 | PAAC26 | **fasgai2** | **PsePC13** | **SP** |
| AAC14 | CTDD39 | **QSO5** | fasgai4 | PsePC14 | **other** |
| **AAC15** | CTDD50 | QSO14 | **hmoment1** | **PsePC16** | **NLS** |
| **APAAC18** | CTDD58 | **QSO18** | instaindex | PsePC17 | TM |
| **APAAC25** | CTDD60 | QSO29 | **pI** | PsePC18 | **SVM_mito** |
| APAAC26 | CTDD67 | **QSO35** | protFP2 | **PsePC19** | **SVM_inter** |
| **APAAC28** | CTDD68 | **QSO39** | **protFP3** | **PsePC20** | **SVM_matrix** |
| APAAC29 | **CTDD71** | QSO41 | protFP7 | **PsePC22** | **Homology** |
| Blosum5 | CTDD82 | QSO44 | **stscales1** | PseSC1 |  |
| Blosum6 | CTDD83 | QSO51 | **stscales5** | **PseSC2** |  |
| Blosum7 | **CTDD92** | QSO52 | tscales5 | **PseSC3** |  |
| **CTDC6** | CTDD93 | QSO54 | **vhsescales3** | **PseSC8** |  |
| **CTDC7** | **CTDD103** | QSO58 | **vhsescales4** | PseSC10 |  |
| **CTDC9** | CTDT4 | SOCN13 | **vhsescales7** | PseSC15 |  |
| CTDC10 | geary3 | SOCN19 | **vhsescales8** | **PseSC19** |  |
| **CTDC17** | **geary5** | **aIndex** | **zscales1** | **Erpred** |  |
| **CTDC20** | geary9 | **autocov** | **zscales2** | **tango1** |  |
| **CTDD1** | geary11 | **crosscov2** | **PsePC2** | tango5 |  |
| CTDD6 | geary17 | crucian3 | PsePC4 | **cTP** |  |
